# Phenothiazine Derivatives: The Importance of Stereoisomerism in the Tolerance and Efficacy of Antimicrobials

**DOI:** 10.1007/s12088-024-01309-3

**Published:** 2024-05-22

**Authors:** Troels Ronco, Maria Juul, Zélie Reynier, Jørn B. Christensen, Søren Svenningsen, Rikke H. Olsen

**Affiliations:** 1 Department of Veterinary and Animal Sciences, Faculty of Health and Medical Sciences, University of Copenhagen, Copenhagen, Denmark; 2 Department of Chemistry, Faculty of Science, University of Copenhagen, Copenhagen, Denmark

**Keywords:** Phenothiazine derivatives, Stereoisomers, Toxicity, Antimicrobial activity, MRSA, In vivo models

## Abstract

Stereoisomers are molecules that are identical in atomic constitution and bonding. The biological properties may, however, differ significantly between two enantiomers (individual stereoisomers). JBC 1847, a phenothiazine derivative with strong antimicrobial activity against Gram-positive bacteria, exists in two enantiomers, S and R. Under standard chemical synthesis (S)-and (R)-JBC 1847 will be present in 50/50 amount (racemic). In this study, we have investigated the antimicrobial activity, the in vivo tolerance and therapeutic efficacy of purified (S)-JBC 1847. Compared to JBC 1847 racemic, the antimicrobial activity of (S)-JBC 1847 in vitro was in the same range or slightly increased, while the maximum tolerable concentration in vivo was five times higher for (S)-JBC 1847 (5 mg/kg versus 20 mg/kg bodyweight). Furthermore, the in vivo efficacy of (S)-JBC 1847 in a mouse peritonitis MRSA model was comparable to the activity of vancomycin. In conclusion, the antimicrobial activity and tolerance of a medical stereoisomeric compound may be significantly different using purified enantiomers compared with the racemic state.

**Supplementary Information:**

The online version contains supplementary material available at 10.1007/s12088-024-01309-3.

## Introduction

During the last two decades, there has been a dramatic increase in antimicrobial resistant (AMR) bacteria causing a serious threat to the human health [1]. This has led to a search for novel antimicrobials in addition to exploring the possibility of re-purposing existing non-antimicrobial drugs as antimicrobials [2]. For the latter, phenothiazines (PHs), traditionally used in psychopharmacology, have been extensively explored as antimicrobials and antimicrobial adjuvants, in particular regarding treatment of infections caused by Methicillin Resistant *Staphylococcus aureus* (MRSA) [3, 4]. Unfortunately, the rapid passage of PHs over the blood–brain-barrier (BBB) has let to severe neurological side effects [5] and general toxicity of PHs have limited their clinical importance as antimicrobials or antimicrobial adjuvants [6]. Previously, we have managed to synthesize a PH derivative, JBC 1847, that do not pass the BBB to any significant degree, possesses up to 256 times higher antimicrobial activity than the original PH promazine and exhibits activity against both planktonic and biofilm-associated bacteria [7, 8]. Chemically, JBC 1847 is a chiral compound that exists in two structure forms (enantiomers), (S)-and (R)-JBC 1847, present in 50/50 amounts (a racemic mixture). Other PHs derivatives, such as thioridazine, are racemic mixtures as well and previously, it has been shown that the pharmacological properties of the two enantiomers can differ extensively [9]. For thioridazine, it has been shown that the (-)-enantiomer binds with lower affinity to the dopamine 2 receptor than the ( +)-thioridazine enantiomer, which could result in decreased cardiotoxic side effects when applied in humans [10]. In addition, the in vitro antimicrobial activity of the (-)-thioridazine enantiomer, was found to be equal to (and in some cases higher) compared to the racemic mixture of thioridazine [4]. (S)-JBC 1847 is equivalent to the (-)-enantiomer of thioridazine, and therefore, we aimed to assess the antimicrobial activity, in vivo tolerance and therapeutic efficacy of (S)-JBC 1847 in this study.

## Materials and Methods

### Bacterial Strains

For the in vitro assessment of the antimicrobial activity of (S)-JBC 1847 a diverse collection of 19 strains of *S. aureus* were chosen (Table S1). Of these, 16 strains were obtained from our in-house strain collection of clinical isolates. Fourteen of the in-house strains were resistant to at least one antimicrobial class, seven were categorized as MRSA strains whereas two strains exhibited no detectable antimicrobial resistance (Table S1). To compare the activity of JBC 1847 (racemic) with (S)-JBC 1847, three well-characterized reference strains of *S. aureus* were chosen for Minimal Inhibition Concentration (MIC) determination. The strains represent a livestock associated MRSA (CC398), a community associated MRSA (USA300) (GenBank: CP020619.1) and the MRSA strain ATCC BAA-1556 (GenBank: CP000255). A vancomycin resistant *Enterococcus* (VRE) strain (*E. faecium* ATCC 700-221) was also included in the assessment. In general, all 19 *S. aureus* strains and the single *E. faecium* strain, were handled as previously described by Ronco et al. [7].

### Purification of (S)-JBC 1847

A racemic mixture of JBC 1847 was synthesized from promazine at The Department of Chemistry, Faculty of Science, University of Copenhagen as previously described [7]. Subsequently, (S)-JBC 1847 was purified from this racemic mixture of JBC 1847 from (S)-Citronellol tosylate (Sigma aldrich, Roskilde, Denmark), prepared according to the protocol published by Shirai et al. 1999 [11].

### In Vitro Analyses of Antimicrobial Activity

Determination of MIC values was performed based on the CLSI guidelines references for the broth dilution method [12]. All MIC assays were run in duplicates and performed as previously described [7].

For determination of MBC, the broth dilution method was performed slightly modified but based on the procedure described by Rodríguez-Melcón et al. [13]. The MBC values were determined for the three MRSA reference strains (CC398, USA300 and ATCC BAA-1556) and the single VRE strain (*E. faecium* ATCC 700-221). Briefly, for MBC determinationa total of 100 μL suspension from each well in the MIC assay was seeded with loop on agar plates (agar base, Sigma, Copenhagen, Denmark) supplemented with 5% bovine blood. The plates were incubated at 37 °C for 48 h. MBC was defined as the lowest concentration of JBC 1847 that reduced the CFU of the original inoculum (1.5 × 105 CFU) by ≥ 99.9%.

### Generation of Mutants with Increased Phenotypically Tolerance to (S)-JBC 1847

The frequency of spontaneous single-step mutations in *S. aureus* and *E. faecium*, respectively, mediating increased tolerance to (S)-JBC 1847, was determined using the gradient plate technique. The plates were prepared in Petri dishes, which were poured with two layers of agar. The bottom layer consisted of Mueller–Hinton agar, allowed to harden with the plate slanted sufficiently to cover the entire bottom. The top layer, added to the dish in the normal position, were supplied with (S)-JBC 1847 to generate concentrations of ∼0.5–8 × MIC through the slope of the plate. An inoculum of 10^9^ CFU of *S. aureus* USA 300 and *E. faecium* ATCC 700–221, respectively, was homogeneously spread on each plate, and incubated for 48 h at 37 °C. Colonies growing at the highest antibiotic concentration were sampled, checked for purity, grown overnight in antibiotic-free broth, and determined for MIC as described above. All experiments were carried out in triplicates.

### Tolerance of (S-) JBC 1847 in a Larvae Model

High concentration tolerance (32 mg/kg) was initially assessed in a *Galleria mellonella* model. The study was performed in accordance to a method previously published by Desbois and Coote, 2011 [14], with minor modification and in technical and biological duplicates.

The larvae were purchased from Monis Pet Store, Viborg, Denmark and upon arrival stored at 5 °C and used within 3 days. The larvae did not receive either food nor water at any timepoint after arrival and were only exposed to light when handled and kept in petri dishes. The dishes were cleaned for fecal discharge and dead larvae were removed every day during the trials. At the end of all trials the larvae were euthanized by freezing to − 20 °C for at least 24 h.

Before injection, the larvae were left to acclimatize for 30 min at room temperature. The larvae were inspected for signs of melanization and larvae with excess darkening of the cuticle were excluded. The larvae weighing between 300 and 400 mg were included in the study only.

The larvae were randomly distributed into four different treatment groups (unexposed control group, vehicle injected, (S)- JBC 1847 (32 mg/kg) and vancomycin injected (32 mg/kg)), each group including 20 larvae.

The larvae were injected intraperitoneally using ‘Insumed’ syringes (Fisher scientific, Roskilde, Denmark) with a 31-gauge needle. The first injection was made by penetrating last proleg on the left side of the larva and the needle was subsequently pushed in cranial direction just beneath the cuticle until the needle-tip reached the adjacent proleg on same side. Subsequent injections were performed the same way only the site of injection was moved one proleg in cranial direction. Following the first injection larvae were placed in petri dishes in an incubator at 37 °C. The larvae received two injections with an interval of 24 h and observations for viability in all groups were observed for 72 h and scored for mortality. Larvae were considered dead and discharged when completely melanized and showed no movement when poked with a needle cap.

A statistical analysis of variance (ANOVA) was used to establish if the groups differed in total mortality.

### Tolerance (S)-JBC 1847 in a Murine Model

Following the compound tolerance evaluation in *G. mellonella*, tolerance to (S-) JBC 1847 and JBC 1847 racemic, respectively, was assessed in a vertebrate (murine) model. The maximum tolerable dosis (MTD) was determined for (S-) JBC 1847 and JBC 1847, respectively by injecting NMRI female mice intraperitoneal (IP) (n = 2) with escalating doses from 0.5 to 25 mg/kg of the compound. The mice were scored for clinical signs of discomfort for 4 h. Mice with no clinical scores within 2 h, were treated with a similar IP dose once again. Mice were scored additionally following 2 h after the last dose. When moderate discomfort was observed the mice were euthanized and the dose was considered “not tolerated”. The MTD for (S)-JBC 1847 and JBC 1847, respectively, were determined as the dose proceeding the “not tolerable” dose.

### Treatment Efficacy in a Murine Peritonitis Model

The treatment efficacy of (S)-JBC 1847 was assessed in a murine peritonitis infection model as previously described by Lundberg et al., 2010 [15], with minor modifications. XX Female NMRI mice (18–22 g) were infected with MRSA 43484 and treated with a dosage of 20 mg/kg (S)-JBC 1847 and treatment efficacy was investigated in two separate assays ((S)-JBC 1847 dissolved in 5% DMSO or β–cycklodextrin). The treatment control group was treated with vancomycin (80 mg/kg) subcutaneously.

## Results

### Antimicrobial Activity of (S)-JBC 1847

**Fig. 1:**
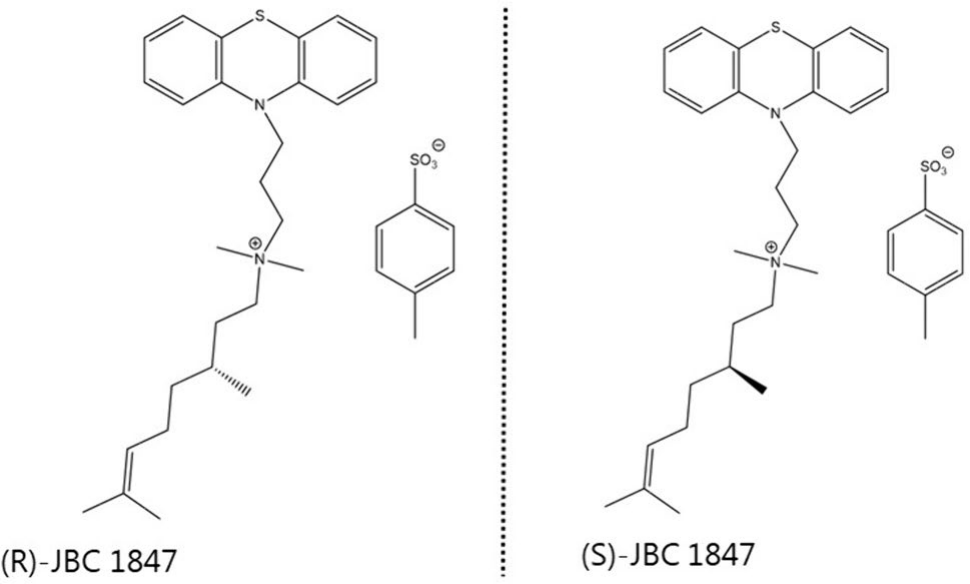
JBC 1847 racemic is composed of two enantiomers, (R)-JBC 1847 and (S)-JBC 1847

(S)- JBC 1847 was successfully synthesized and represents one of the two enantiomers of JBC 1847 racemic (Fig. 1), and subsequently, the antimicrobial activity of (S)-JBC 1847 was assessed.

The MIC values varied between 1–2 mg/L, while the MBC values varied between 1 and 4 mg/L, depending on species (Table 1). For all evaluated isolates of *S. aureus*, the MIC equaled the MBC, while for *E. faecium* MBC was twofold higher than the MIC.

**Table 1:** Minimum inhibitory concentration (MIC) and Minimum bactericidal concentrations (MBC) of (S)-JBC 1847 for selected reference strains

Strain	MIC (mg/L)	MBC (mg/L)
MRSA CC398	1	1
MRSA USA300	1	1
MRSA ATCC BAA-1556	1	1
VRE ATCC 700-221	2	4

In addition, 16 *S. aureus* in-house strains with diverse resistance profiles were tested against (S)-JBC 1847. The MIC values of these strains ranged from 0.125 to 1 mg/L (Table S1). There was no notable difference between the MIC values of the strains resistant to commonly used antimicrobials compared to the two fully susceptible strains (Table S1). Thereby, no remarkable difference in MIC values between the four reference strains tested against JBC 1847 and the 16 in-house strains tested against (S)-JBC 1847, was observed.

### Tolerance of (S-)JBC 1847 in *Galleria mellonella* Larvae

A preliminary screening of the toxic potential of (S)-JBC 1847 was performed using *G. mellonella* larvae. Mortality after 72 h was scored for each group (Table 2). Statistical analysis of variance (ANOVA) revealed no difference in mortality between the four groups included in the study (unexposed group, vehicle treatment group, (S)-JBC 1847 treatment group and vancomycin treatment group) (*p* = 0.123).

**Table 2:** Tolerance study of (S)-JBC 1847 in Galleria mellonella larvae

		Unexposed	Vehicle	(S)-JBC 1847 1120 mg/L	Vancomycin 1120 mg/L
Trial 1	Alive	18 (90%)	17 (85%)	13 (65%)	19 (95%)
	Dead	2 (10%)	3 (15%)	7 (35%)	1 (5%)
	Mortality risk	0.1	0.15	0.35	0.05
Trial 2	Alive	19 (95%)	17 (85%)	16 (80%)	17 (85%)
	Dead	1 (5%)	3 (15%)	4 (20%)	3 (15%)
	Mortality risk	0.05	0.15	0.2	0.15

### Establishment of MTD in Mice

For mice treated with JBC 1847 racemic, moderate signs of discomfort (incl either/or blue tails, piloerection, separation from cage mates etc.) occurred when dosed at10 mg/kg, hence the MTD at IP administration was 5 mg/kg (one dosing level under 10 mg/kg), while for (S)-JBC 1847 no clinical signs of discomfort occurred until dosing reached 25 mg/kg,

### Efficacy of (S)-JBC 1847 in a Murine Model

**Fig. 2:**
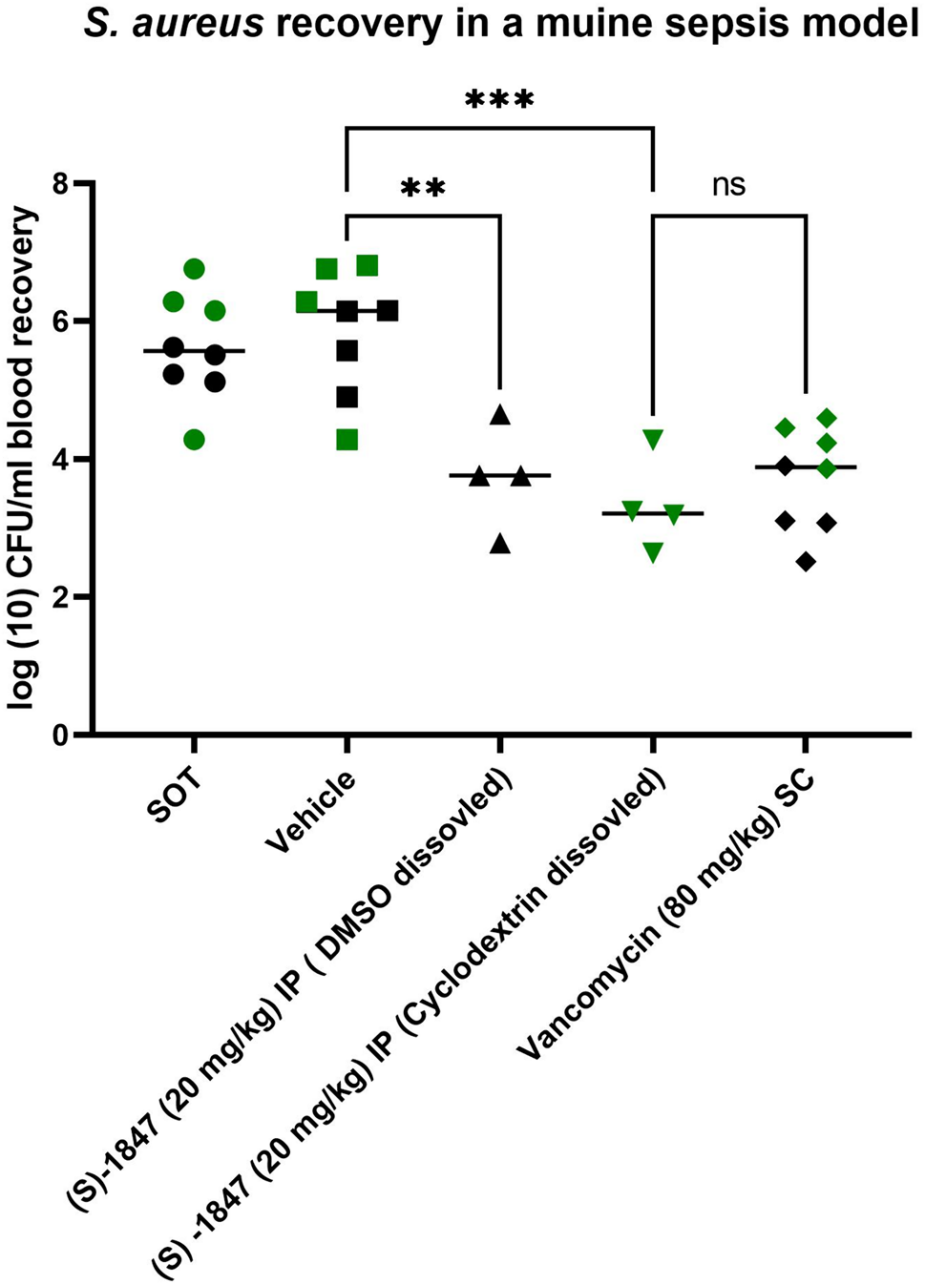
Treatment efficacy of (S)-JBC 1847 in mice inoculated with MRSA 43484. Efficacy was measured as reduction of colony forming unit (CFU) per ml in (S)-JBC 1847 treatment group compared to vehicle treated

The in vivo efficacy of (S)-JBC 1847 was investigated in a murine MRSA peritonitis infection model. The results in Fig. 2 showed that the activity of (S)-JBC 1847 was comparable to the activity of vancomycin, measured by reduction in colony forming units (CFU)/ml blood compared to level at start of treatment (SOT).

## Discussion

Two enantiomers of the same compound can exhibit extensively variations in biological activity and thus, stereochemical considerations are important in regard to design of novel antimicrobial agents [16, 17]. High antimicrobial activity of JBC 1847 has been demonstrated [7, 8], but all previous studies of JBC 1847 has been carried out using a racemic mixture consisting of (R)- and (S)- JBC 1847.Thus, we aimed to study the properties of the purified (S)-enantiomer of JBC 1847.

The antimicrobial activity of (S)-JBC 1847 for Gram-positive was in the same range as previously reported for the racemic of JBC 1847 (Table 1) [7]. Notably, the antimicrobial activity of (S)-JBC did not depend on the genetic resistance background of the strain, as equal activity was observed for high (conventional) antimicrobial resistant strains as for fully (conventional) antimicrobial susceptible strains (Table 1). Hence, for the resistances presented in strains in this study, there was no indication of cross-resistance between (S)-JBC 1847 and other antibiotic resistance mechanisms towards conventional antimicrobials. In line with this, no stable phenotype with increased tolerance to (S)-JBC 1847 for either *S. aureus* or *E. faecium* could be generated. Therefore, if bacterial resistance (or increased tolerance) against JBC 1847 should develop, this will most likely be a multistep process or depending on induction of other factor, such as increased efflux. We have previously shown that induction of resistance seems very difficult for JBC 1847 racemic, but if the antimicrobial mode of action is the same for (S)-JBC 1847 as it is for JBC 1847 racemic has not yet been explored although this study has documented that (S)-JBC 1847 acts as a bactericidal antimicrobial similar to the racemic (Table 1) [7].

Pharmaceuticals used for treatment of disease in humans and animals must show low toxicity and be safe to use even in doses above the therapeutical index [18]. Therefore, a tolerance study of (S)-JBC 1847 was carried out in *G. mellonella* larva which has previously been shown to represent a valid model for prediction of drug toxicity in humans [19, 20]. The mortality risk in the (S)-JBC 1847 was not found to be significantly different from the mortality risk in any of the control groups (Table 2). As there was no difference in mortality risk between the unexposed group and the injected groups, it could be concluded that (S)-JBC in high concentrations was well tolerated in the non-vertebrate model. The tolerance of (S)-JBC 1847 was confirmed in a mammalian study, in which no signs of acute toxicity was observed tolerated when dosing mice in a range up to 20 mg/kg. Appling this dose for treatment of IP MRSA inoculated mice, we were able to demonstrate a treatment efficacy of (S)-JBC 1847 comparable to treatment with vancomycin, a standard conventional antibiotic for treatment of MRSA infections As the racemic of JBC 1847 was tolerated very poorly in the initial safety screening, further in vivo studies with the racemic were discontinued.

## Conclusion

Compared to JBC 1847 racemic, the antimicrobial activity of the (S)-enantiomer of JBC 1847 was not compromised, while the in vivo tolerance was greatly improved, and a therapeutic effect of MRSA infected mice was observed. Further in-deep toxicological examinations on organ level and after prolonged time of use, are needed to fully verify the potential of (S)-JBC 1847 as a new antimicrobial. Nevertheless, results of this study encourage greater attention to the potential of using single enantiomers rather than racemic mixtures when assessing chiral medical compounds.

### Supplementary Information

Below is the link to the electronic supplementary material.

Supplementary file1 (DOCX 18 kb)
