# Supplementary material for: Phenothiazine Derivatives: The Importance of Stereoisomerism in the Tolerance and Efficacy of Antimicrobials
Source: Indian J Microbiol. 2024 May 22;64(2):743–8. doi: 10.1007/s12088-024-01309-3 (PMC11246383; doi:10.1007/s12088-024-01309-3)
Supplement: Supplementary file 1 — Supplementary file1 (DOCX 18 kb) [file 12088_2024_1309_MOESM1_ESM.docx]

**Table S1** Minimum inhibitory concentration (MIC) of (S)-JBC 1847 against selected *Staphylococcus aureus*

| Strain | Resistance | MIC |
| --- | --- | --- |
| C-22356- 2 | Ampicillin, Clindamycin  Imipenem, Penicillin, Tetracyclin | 0.5 |
| C-23875 | Amoxicillin/clavulanic acid, Ampicillin, Imipenem, Penicillin, Sulfamethoxazole-  Trimethoprim | 0.25 |
| C-23964- 1 | Amoxicillin/clavulanic acid, Ampicillin, Imipenem, Penicillin, Sulfamethoxazole-  Trimethoprim | 8 |
| C-23964- 1 | Amoxicillin/clavulanic acid, Ampicillin, Imipenem, Penicillin, Sulfamethoxazole-  Trimethoprim | 4 |
| C-23909 | Clindamycin, Enrofloxacin,  Erythromycin | 0.5 |
| C-24715 | Ampicillin, Clindamycin,  Erythromycin, Imipenem,  Penicillin | 0.5 |
| C-24760 | Amikacin, Ampicillin,  Gentamicin, Imipenem, Penicillin | 0.25 |
| C-24937 | Amoxicillin/clavulanic acid, Chloramphenicol, Tetracyclin, Sulfamethoxazole-  Trimethoprim | 0.25 |
| C-25054 | Amoxicillin/clavulanic  acid, Ampicillin,  Cefazolin, Cefoxitin, | 0.5 |

|  | Cefpodoxime, Ceftiofur, Cephalothin, Clindamycin, Enrofloxacin, Erythromycin, Imipenem, Marbofloxacin, Orbifloxacin, Oxacillin, Penicillin, Ticarcillin,  Ticarcillin/clavulanic acid |  |
| --- | --- | --- |
| C-29630 | Amoxicillin/clavulanic acid, Ampicillin, Cefazolin, Cefovecin, Cefoxitin, Cefpodoxime, Ceftiofur, Imipenem, Oxacillin, Penicillin, Ticarcillin,  Ticarcillin/clavulanic acid | 0.25 |
| C-30935 | Amoxicillin/clavulanic acid, Ampicillin, Cefazolin, Cefazolin, Cefovecin, Cefoxitin, Cefpodoxime, Oxacillin,  Penicillin | 0.5 |
| C-31534 | Amoxicillin/clavulanic acid, Ampicillin, Cefazolin, Cefovecin, Cefoxitin, Cefpodoxime, Ceftiofur, Marbofloxacin,  Oxacillin, Penicillin | 0.5 |
| C-31598 | Amoxicillin/clavulanic acid, Ampicillin, Cefazolin, Cefovecin, Cefoxitin, Cefpodoxime, Ceftiofur, Clindamycin, Erythromycin, Marbloflox, Oxacillin,  Penicillin | 0.5 |
| C-31667 | Amikacin, Amoxicillin/clavulanic  acid, Ampicillin, | 0.25 |

|  | Cefazolin, Cefovecin, Cefoxitin, Cefpodoxime, Clindamycin, Erythromycin, Oxacillin, Penicillin |  |
| --- | --- | --- |
| C-31781 | Amikacin, Amoxicillin/Clavulanic acid, Ampicillin, Cefazolin, Cefovecin, Cefoxitin, Cefpodoxime,  Ceftiofur, Gentamicin, | 0.5 |
|  | Marbofloxacin, Oxacillin,  Penicillin |  |
| C-45649 | Amoxicillin/clavulanic acid, Ampicillin, Cefazolin, Cefovecin, Cefpodoxime,  Cephalothin, | 0.25 |
|  | Clindamycin, Imipenem,  Oxacillin, Penicillin, Rifampin |  |
